# Supplementary material for: Transcriptome and Degradome Profiling Reveals a Role of miR530 in the Circadian Regulation of Gene Expression in Kalanchoë marnieriana
Source: Cells. 2021 Jun 17;10(6):1526. doi: 10.3390/cells10061526 (PMC8233840; doi:10.3390/cells10061526)
Supplement: Supplementary file 1 [file cells-10-01526-s001.zip › SuppleInfo_figs_tables_rev1.pdf]

Supplementary information

Supplementary figures

Supple. Figure 1. ***K. marnieriana*** **is a constitutive CAM plants with enhanced leaf titratable acids under drought treatments**. **A**, an overall morphology of a *K. marnieriana* plant maintained in a growth chamber. **B**, the phylogenetic relationship of closely related *K. marnieriana* plants based on ITS sequences analysis [1]. KM "BaiJiZhiWu" is the accession used in this study. **C**, the measurements of leaf titratable acid under regularly watering condition (CK), withhold watering for 10 days (D10) and withhold watering for 25 days (D25).

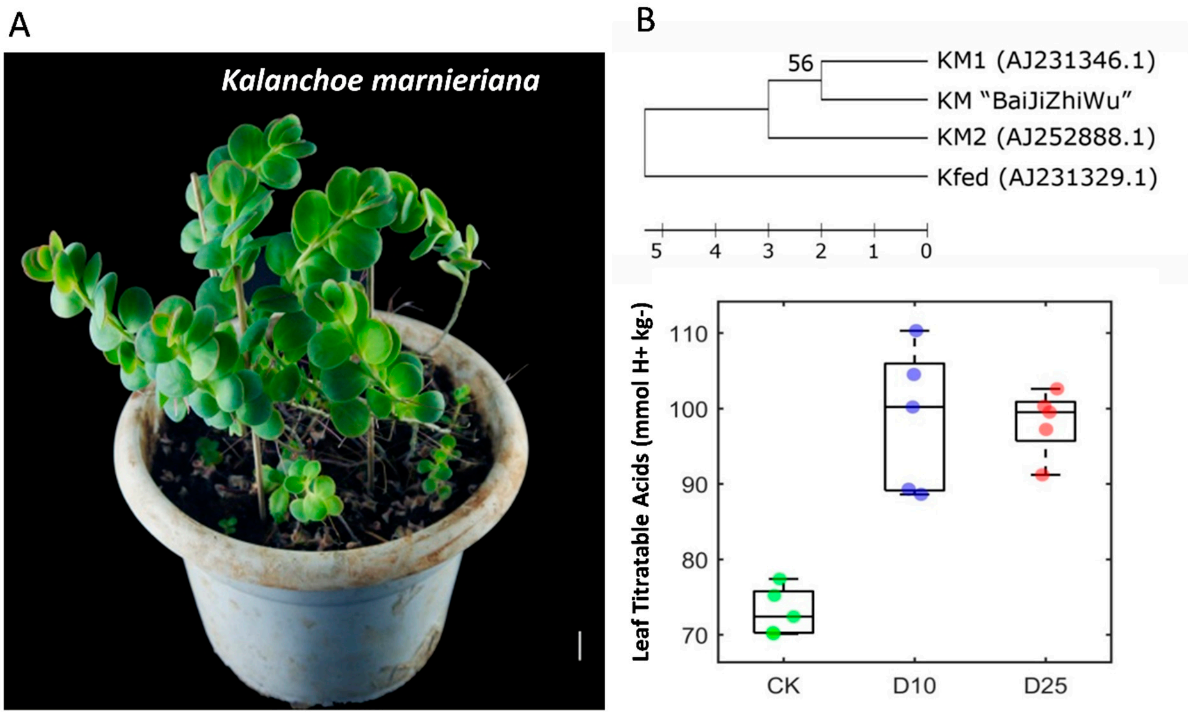

Supple. Fig2. **Diurnal expression of potential miRNA targets that are identified by the degradome analysis**. **A**, a heatmap plot of diel expression of targets. The groups of morning-specific and dawn-specific expression genes are highlighted by black boxes. **B**, The TZP-like genes (TZP1 and TZP2) are revealed as the morning-specific genes that are targeted by miR530. Kal-miR_C1817 is revealed to target POF and MYB genes.

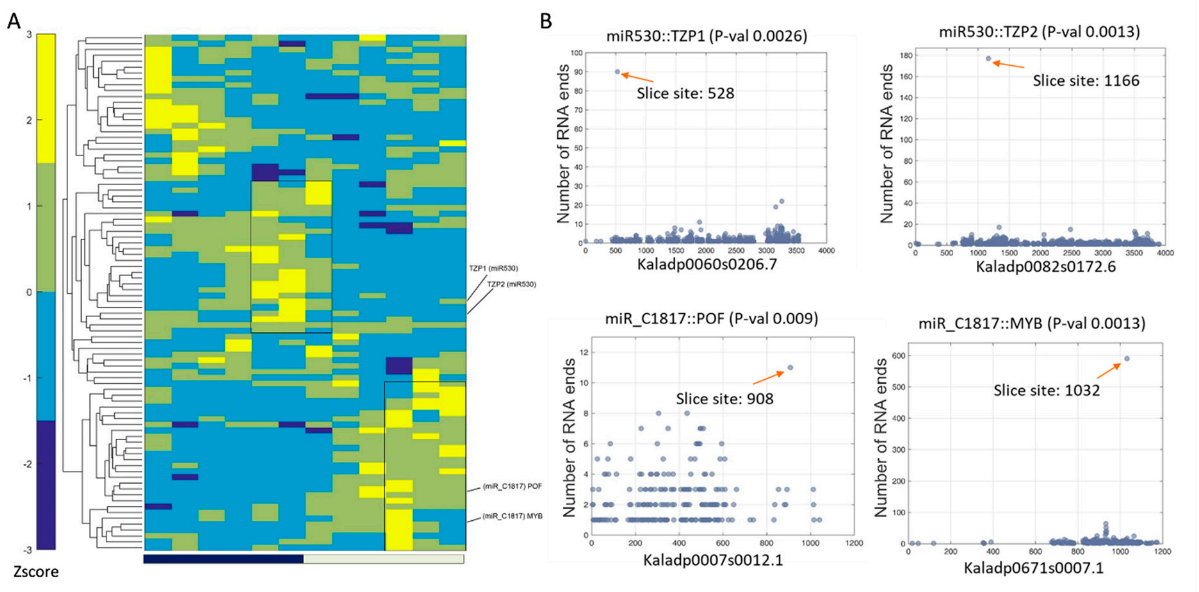

Supple. Table S1. The primers used in this study.

Primer name	Sequences	Usage
**TEDI-F**	TGTTGTTCCTGCCACAGAAG	Internal control realtime PCR
**TEDI-R**	TAGAGGGTGAAGGTCCCAGA	Internal control realtime PCR
**TZP1-F**	TTAGCCGGTAAAAGCTACCATGTT	realtime PCR
**TZP1-R**	TGGAACCAGCTTGCTCATTTG	realtime PCR
**TZP2-F**	TTTATCATGTGGCGTACGTTGAT	realtime PCR
**TZP2-R**	AGAACAAGCTTGGCACCTTTAGA	realtime PCR
**mi530-mature**	TGCATTTGCACCTGCACCTTT	realtime PCR
**pre-mi530+EX-F**	CTCGAGCTTACCCTCCTCCTTC	miRNA over-expressor
**pre-mi530+EX-R**	GAATTCCCACTGCACAGACAC	miRNA over-expressor
**TZP1-F**	CTAGGTTAGGTGCAGGTGTAAATGCAA	Dual-Luciferase Reporters
**TZP1-R**	CCGGTTGCATTTACACCTGCACCTAAC	Dual-Luciferase Reporters
**TZP2-F**	CTAGGTCAGGCGCAGGTGCAAATGCAA	Dual-Luciferase Reporters
**TZP2-R**	CCGGTTGCATTTGCACCTGCGCCTGAC	Dual-Luciferase Reporters

Supple. Table S2. The statistics of small RNA sequencing results in *Kalanchoë marianeria*

The number of reads were filtered using

	**Total**	**Percentage**	**Unique**	**Percentage**
Raw reads	251741931	100.00%	106659931	100.00%
3ADT&length filter	34244478	13.60%	13432958	12.59%
Junk reads	1116037	0.44%	710834	0.67%
Rfam	10230068	4.06%	158387	0.15%
Repeats	118538	0.05%	3212	0.00%
valid reads	206077270	81.86%	92355587	86.59%
rRNA	9146690	3.63%	126123	0.12%
tRNA	490550	0.19%	11605	0.01%
snoRNA	63505	0.03%	4281	0.00%
snRNA	62392	0.02%	3979	0.00%
other Rfam RNA	466931	0.19%	12399	0.01%

Supple. Table 3. The predict miRNA targets in the diel transcriptome based on the degradome analysis. The slice site and the alignment are identified by the CleaveLand 4.0 pipeline (https://github.com/MikeAxtell/CleaveLand4).

Transcript Name	Slice Site	Query(miRNA)	Category	p-value	Arabidopsis-symbol
Kaladp0078s0017.1	869	kal-miR164d	0	0.00043	anac021,ANAC022,NAC1
Kaladp0002s0088.1	1899	mdm-miR160a	0	0.00043	ARF16
Kaladp0072s0008.1	3436	mdm-miR162a	0	0.00043	ASU1,ATDCL1,CAF,DCL1,EMB60,EMB76,SIN1,SUS1
Kaladp0099s0085.3	1725	mdm-miR172i_R-1	0	0.00043	RAP2.7,TOE1
Kaladp0891s0016.1	1750	mdm-MIR172i-p5	0	0.00043	AP2,FL1,FLO2
Kaladp0024s0700.1	699	mdm-miR2111a	0	0.00043	
Kaladp0058s0662.1	3179	mdm-miR403a	0	0.00043	AGO2
Kaladp0007s0012.1	908	kal-miR_C1878_star	0	0.00086	
Kaladp0192s0039.1	1060	kal-miR164d	0	0.00086	ANAC098,ATCUC2,CUC2
Kaladp0095s0509.1	1300	kal-miR166a	0	0.00086	ATHB-15,ATHB15,CNA,ICU4
Kaladp0032s0311.1	1525	kal-miR172c	0	0.00086	RAP2.7,TOE1
Kaladp0381s0002.1	300	kal-miR858	0	0.00086	AtMYB6,MYB6
Kaladp0964s0014.1	950	mdm-miR159d	0	0.00086	ATMYB65,MYB65
Kaladp0002s0088.2	1515	mdm-miR160a	0	0.00086	ARF16
Kaladp0092s0159.1	1341	mdm-miR166a	0	0.00086	ATHB-15,ATHB15,CNA,ICU4
Kaladp0053s0463.1	686	mdm-miR168a_1ss2CT	0	0.00086	AGO1
Kaladp0032s0311.3	1495	mdm-miR172a_R+1	0	0.00086	RAP2.7,TOE1
Kaladp0099s0085.2	1728	mdm-miR172i_R-1	0	0.00086	RAP2.7,TOE1

Kaladp0099s0085.1	1550	mdm-MIR172i-p5	0	0.00086	RAP2.7,TOE1
Kaladp0024s0700.2	560	mdm-miR2111a	0	0.00086	
Kaladp0094s0012.1	1656	mdm-miR156t_L-1_1ss15AT	1	0.001	
Kaladp0671s0007.1	1032	kal-miR_C1878_star	0	0.00129	ATMYB66,MYB66,WER,WER1
Kaladp0048s0186.1	867	kal-miR164d	0	0.00129	ANAC100,ATNAC5,NAC100
Kaladp0099s0085.4	1674	mdm-MIR172i-p5	0	0.00129	RAP2.7,TOE1
Kaladp0515s0205.1	301	mdm-MIR858-p5_1ss17AG	0	0.00129	ATMYB5,MYB5
Kaladp0082s0172.6	1166	ptc-miR530a_R+1_1ss20TG	0	0.00129	TZP
Kaladp0674s0167.1	889	kal-miR164d	0	0.00172	ANAC079,ANAC080,ATNAC4,NAC080
Kaladp0043s0276.1	1291	kal-miR166a	0	0.00172	ATHB-15,ATHB15,CNA,ICU4
Kaladp0840s0019.1	1865	kal-miR319	0	0.00172	ATTCP24,TCP24
Kaladp0058s0602.1	739	lja-miR397_L-2R+2	0	0.00172	ATLAC17,LAC17
Kaladp0040s0160.1	1321	mdm-miR156a_1ss16AG	0	0.00172	SPL2
Kaladp0087s0171.1	969	mdm-miR156t_L-1R+1_1ss15AT	0	0.00172	SPL9
Kaladp0008s0410.1	1782	mdm-miR160a	0	0.00172	ARF16
Kaladp0032s0311.2	1450	mdm-miR172a_R+1	0	0.00172	RAP2.7,TOE1
Kaladp0082s0172.1	1317	ptc-miR530a_R+1_1ss20TG	0	0.00172	TZP
Kaladp0095s0553.1	863	kal-miR164d	0	0.00215	ANAC100,ATNAC5,NAC100
Kaladp0008s0827.1	1161	kal-miR166a	0	0.00215	ATHB-14,ATHB14,PHB,PHB-1D
Kaladp0101s	1649	kal-miR172c	0	0.00215	RAP2.7,TOE1

0311.2					
Kaladp0003s 0028.1	803	lja-miR397_L-2R+2	0	0.00215	LAC12
Kaladp0088s 0066.1	1536	mdm-miR156a_1ss16AG	0	0.00215	SPL2
Kaladp0011s 1053.1	843	mdm-miR156t_L-1R+1_1ss15AT	0	0.00215	
Kaladp0039s 0575.1	1601	mdm-miR160a	0	0.00215	ARF16
Kaladp0101s 0311.1	1694	mdm-miR172a_R+1	0	0.00215	RAP2.7,TOE1
Kaladp0071s 0243.2	1751	mdm-miR172i_R-1	0	0.00215	RAP2.7,TOE1
Kaladp0082s 0172.3	1196	ptc-miR530a_R+1_1ss20TG	0	0.00215	TZP
Kaladp0039s 0699.2	812	kal-miR166a	0	0.00258	IFL,IFL1,REV
Kaladp0060s 0206.7	528	kal-miR530a	0	0.00258	TZP
Kaladp0003s 0134.1	649	kal-miR858	0	0.00258	ATMYB12,MYB12,PFG1
Kaladp0039s 0575.2	1490	mdm-miR160a	0	0.00258	ARF16
Kaladp0071s 0243.1	1744	mdm-miR172i_R-1	0	0.00258	RAP2.7,TOE1
Kaladp0003s 0134.3	800	mdm-MIR858-p5_1ss17AG	0	0.00258	ATMYB12,MYB12,PFG1
Kaladp0082s 0172.2	1233	ptc-miR530a_R+1_1ss20TG	0	0.00258	TZP
Kaladp0059s 0285.1	261	ath-MIR858a-p5_1ss17AG	0	0.00301	
Kaladp0008s 0827.3	1028	kal-miR166a	0	0.00301	ATHB-14,ATHB14,PHB,PHB-1D
Kaladp0060s 0206.6	639	kal-miR530a	0	0.00301	TZP
Kaladp0024s 0285.2	1196	mdm-miR159d	0	0.00301	ATMYB65,MYB65
Kaladp0033s 0012.1	646	mdm-miR160a	0	0.00301	ARF10
Kaladp0081s	1749	mdm-miR166a	0	0.00301	IFL,IFL1,REV

0257.1					
Kaladp0082s 0172.5	838	ptc-miR530a_R+1_1ss20TG	0	0.00301	TZP
Kaladp0060s 0206.5	528	kal-miR530a	0	0.00344	TZP
Kaladp0003s 0134.2	1084	kal-miR858	0	0.00344	
Kaladp0024s 0285.1	2322	mdm-miR159d	0	0.00344	ATMYB65,MYB65
Kaladp0039s 0699.1	1611	mdm-miR166a	0	0.00344	IFL,IFL1,REV
Kaladp0082s 0172.7	908	ptc-miR530a_R+1_1ss20TG	0	0.00344	TZP
Kaladp0023s 0071.1	1071	kal-miR396b	0	0.00387	AtGRF1,GRF1
Kaladp0060s 0206.1	1624	kal-miR530a	0	0.00387	TZP
Kaladp0003s 0134.4	993	kal-miR858	0	0.00387	
Kaladp0042s 0136.1	2093	mdm-miR159d	0	0.00387	ATMYB65,MYB65
Kaladp1184s 0001.1	1364	mdm-miR160a	0	0.00387	ARF17
Kaladp0081s 0257.2	1666	mdm-miR166a	0	0.00387	IFL,IFL1,REV
Kaladp0082s 0172.4	908	ptc-miR530a_R+1_1ss20TG	0	0.00387	TZP
Kaladp0023s 0071.2	529	kal-miR396b	0	0.0043	AtGRF1,GRF1
Kaladp0060s 0206.3	405	kal-miR530a	0	0.0043	TZP
Kaladp0042s 0136.2	1098	mdm-miR159d	0	0.0043	ATMYB65,MYB65
Kaladp0048s 0026.1	1910	mdm-miR160a	0	0.0043	ARF16
Kaladp0011s 0278.2	1270	kal-miR396b	0	0.00473	AtGRF3,GRF3
Kaladp0060s 0206.4	1624	kal-miR530a	0	0.00473	TZP
Kaladp0061s 0059.1	1734	mdm-miR160a	0	0.00473	ARF16

Kaladp0040s 0154.2	986	kal-miR156a	0	0.00515	
Kaladp0040s 0154.4	1189	kal-miR156a	0	0.00515	
Kaladp0011s 1278.1	1152	kal-miR396b	0	0.00515	AtGRF7,GRF7
Kaladp0060s 0206.2	414	kal-miR530a	0	0.00515	TZP
Kaladp0040s 0154.7	1082	mdm-miR156t_L-1_1ss15AT	0	0.00515	
Kaladp0008s 0827.2	1057	mdm-miR166a	0	0.00515	ATHB-14,ATHB14,PHB,PHB-1D
Kaladp0131s 0018.1	729	mdm-miR2111a	0	0.00515	
Kaladp0040s 0411.1	464	kal-miR_C1878_star	0	0.00558	ATMYB66,MYB66,WER,WER1
Kaladp0040s 0154.3	1125	kal-miR156a	0	0.00558	
Kaladp0040s 0154.5	1094	kal-miR156a	0	0.00558	
Kaladp0053s 0048.1	1702	kal-miR167a	0	0.00558	ARF6
Kaladp0011s 0278.1	1270	kal-miR396b	0	0.00558	AtGRF4,GRF4
Kaladp0008s 0813.1	2639	kal-miR167a	0	0.00601	ARF8,ATARF8
Kaladp0630s 0030.1	738	kal-miR397d	0	0.00601	ATLMCO4,IRX12,LAC4,LMCO4
Kaladp0131s 0018.2	725	mdm-miR2111a	0	0.00601	
Kaladp0039s 0128.1	922	ppe-miR169d_L-1R+1_1ss2GC	0	0.00601	
Kaladp0008s 0921.1	206	kal-miR172c	0	0.00644	
Kaladp0042s 0376.1	1113	kal-miR396b	0	0.00644	AtGRF7,GRF7
Kaladp0040s 0154.6	1988	mdm-miR156t_L-1_1ss15AT	0	0.00644	
Kaladp0023s 0076.1	239	mdm-miR2111a	0	0.00644	XTH6
Kaladp0911s 0004.2	915	kal-miR166a	0	0.00687	ABI5,GIA1

Kaladp1040s0002.2	1152	kal-miR167a	0	0.00687	ARF6
Kaladp0008s0921.2	206	kal-miR172c	0	0.00687	
Kaladp0032s0085.1	976	kal-miR396b	0	0.00687	AtGRF5,GRF5
Kaladp0011s0430.1	422	kal-miR858	0	0.00687	ATMYB4,MYB4
Kaladp1040s0002.1	1224	kal-miR167a	0	0.00729	ARF6
Kaladp0911s0004.1	1001	mdm-miR166a	0	0.00729	ABI5,GIA1
Kaladp0011s0687.1	916	mdm-miR2111a	0	0.00729	
Kaladp0011s1344.1	1955	kal-miR319	0	0.00772	MEE35,TCP4
Kaladp0630s0020.1	1741	kal-miR319	0	0.00815	MEE35,TCP4
Kaladp0017s0020.1	1491	mdm-miR156t_L-1_1ss15AT	0	0.00815	
Kaladp0050s0212.1	644	kal-miR396b	0	0.00858	AtGRF2,GRF2
Kaladp0017s0020.3	1377	mdm-miR156t_L-1_1ss15AT	0	0.00858	
Kaladp0045s0344.1	2578	kal-miR167a	0	0.009	ARF8,ATARF8
Kaladp0008s0428.1	159	kal-miR172c	0	0.009	
Kaladp0017s0020.2	1172	mdm-miR156t_L-1_1ss15AT	0	0.009	
Kaladp0011s0794.1	1794	ppe-miR169d_L-1R+1_1ss2GC	0	0.009	ATHAP2A,EMB2220,HAP2A,NF-YA1
Kaladp0045s0344.2	2140	kal-miR167a	0	0.00943	ARF8,ATARF8
Kaladp0044s0073.1	487	kal-miR858	0	0.01071	ATMYB111,MYB111,PFG3
Kaladp0062s0155.1	1768	mdm-miR160a	0	0.01071	NPY2
Kaladp0441s0001.2	1216	kal-miR858	0	0.01284	ATMYB123,ATTT2,MYB123,TT2
Kaladp0441s	1130	kal-miR858	0	0.01326	ATMYB12,MYB12,PFG1

0001.1					
Kaladp0082s 0106.1	174	mtr-MIR2673a-p3_2ss3TA20TG	0	0.01666	ATFIB2,FIB2
Kaladp0011s 1345.4	932	mdm-miR159d	1	0.01863	
Kaladp0066s 0109.1	465	kal-miR858	0	0.01877	ATMYB5,MYB5
Kaladp0096s 0027.1	270	kal-miR858	1	0.02099	ATMYB3,MYB3
Kaladp0020s 0191.2	645	kal-miR396b	0	0.02844	WRKY21
Kaladp0192s 0023.1	561	mdm-miR396b_1ss2TC	0	0.0297	ATMMS21,HPY2,MMS21
Kaladp0674s 0155.1	1443	kal-miR167a	0	0.03095	AtTCP14,TCP14
Kaladp0032s 0387.1	241	mtr-MIR2673a-p3_2ss3TA20TG	0	0.03095	
Kaladp0043s 0146.1	304	kal-miR_C689	0	0.0347	PQL1,PQL2
Kaladp0630s 0021.1	1263	mdm-miR159d	0	0.03677	
Kaladp0011s 1345.1	1422	mdm-miR159d	0	0.0376	
Kaladp0011s 1345.3	1234	mdm-miR159d	0	0.03802	
Kaladp0030s 0093.3	533	kal-miR166a	0	0.03885	AtTLP3,TLP3
Kaladp0878s 0004.1	154	fve-miR396e_R+1_1ss21TC	1	0.03942	
Kaladp0030s 0093.2	486	kal-miR166a	0	0.04009	AtTLP3,TLP3
Kaladp0030s 0093.1	662	kal-miR166a	0	0.0405	AtTLP3,TLP3
Kaladp0213s 0007.1	942	kal-miR166a	0	0.04339	GAMMA-SNAP,GSNAP
Kaladp0037s 0134.1	503	kal-miR858	0	0.04627	AtMYB109,MYB109
Kaladp0168s 0012.1	815	mdm-miR159d	0	0.04627	
Kaladp0024s 0331.2	1421	ppe-miR169d_L-1R+1_1ss2GC	0	0.04709	NF-YA9
Kaladp0058s	1253	mdm-	1	0.04918	

0159.1		miR393g_2ss12 CT16TC			
Kaladp0013s 0034.1	300	kal-miR858	0	0.05282	ATMYB73,MYB73
Kaladp0779s 0001.2	217	ath-MIR858a-p5_1ss17AG	4	0.05487	
Kaladp0048s 0300.1	1742	kal-miR_C450_star	4	0.05487	
Kaladp0024s 0105.1	233	kal-miR_C5376	4	0.05487	
Kaladp0002s 0088.2	1516	mdm-miR160a_L+1R-1	4	0.05487	ARF16
Kaladp0040s 0342.1	175	kal-miR_C625_star	1	0.06278	PMP22
Kaladp0100s 0087.8	1370	kal-miR858	1	0.06503	
Kaladp0045s 0167.1	1018	bol-MIR9410-p3_2ss5TG18TA	2	0.06702	ATMGL,MGL
Kaladp0048s 0753.2	252	cca-miR396a-3p_L+2R-2	2	0.06702	
Kaladp0007s 0012.1	854	kal-miR_C1878	2	0.06702	
Kaladp0007s 0012.2	986	kal-miR_C1878_star	2	0.06702	
Kaladp0024s 0285.2	1195	kal-miR_C207_star	2	0.06702	ATMYB65,MYB65
Kaladp0071s 0070.1	457	kal-miR_C400_star	2	0.06702	
Kaladp0087s 0188.1	972	kal-miR167a_star	2	0.06702	
Kaladp0779s 0001.1	251	kal-miR858	2	0.06702	
Kaladp0087s 0171.1	970	mdm-miR156t	2	0.06702	SPL9
Kaladp0093s 0080.1	1547	mdm-miR171c	2	0.06702	ATHAM3,HAM3
Kaladp0032s 0361.1	1008	mdm-miR171c_1ss14 CT	2	0.06702	ATHAM3,HAM3
Kaladp0099s 0085.3	1726	mdm-miR172i_L+1R-1	2	0.06702	RAP2.7,TOE1
Kaladp0007s 0058.2	180	vvi-MIR3627-p5_2ss5CG18C	2	0.06702	ELF9

		A			
Kaladp0024s 0457.1	589	kal-miR_C689_star	3	0.07181	
Kaladp0067s 0186.1	1151	kal-miR319_star	3	0.07181	
Kaladp0048s 0753.2	250	kal-miR396b_star	3	0.07181	
Kaladp0550s 0075.1	1294	kal-miR530a_star	3	0.07181	
Kaladp0098s 0188.1	2417	mtr-miR396b-3p_2ss5AG20AT	3	0.07181	AHA2,HA2,PMA2
Kaladp0002s 0132.1	955	csi-MIR399f-p5_1ss8TC	1	0.07914	iqd2
Kaladp0067s 0202.2	510	mdm-miR171c	0	0.08055	
Kaladp0057s 0041.1	2846	kal-miR166a_star	0	0.0845	
Kaladp0024s 0280.3	548	kal-miR_C625_star	0	0.08765	
Kaladp0102s 0075.1	1443	mdm-miR159d	1	0.08958	AP4.3A
Kaladp0079s 0026.1	236	kal-miR397d	0	0.09039	
Kaladp0001s 0197.2	303	kal-miR156a_star	0	0.09235	
Kaladp0063s 0058.1	2178	gma-MIR5775-p3_2ss9AC18GT	0	0.09547	CRR2
Kaladp0008s 0391.1	391	kal-miR858	0	0.10323	ATMYB7,ATY49,MYB7
Kaladp0008s 0500.2	556	kal-miR858	0	0.104	ATMYB123,ATTT2,MYB123,TT2
Kaladp0035s 0040.1	617	kal-miR396b	0	0.10439	
Kaladp0008s 0500.3	467	kal-miR858	0	0.10593	ATMYB123,ATTT2,MYB123,TT2
Kaladp0008s 0500.1	412	kal-miR858	0	0.1067	ATMYB12,MYB12,PFG1
Kaladp0068s 0375.2	295	kal-miR_C689_star	4	0.10673	HXK3
Kaladp0102s 0152.2	1945	kal-miR156a_star	4	0.10673	
Kaladp0072s 0073.3	406	lus-MIR399b-p5_2ss6AG17AT	4	0.10673	

Kaladp0011s0996.1	364	mdm-miR403a	4	0.10673	
Kaladp0036s0147.1	2933	ppe-MIR171d-p3_1ss13TG	4	0.10673	TPR4,WSIP2
Kaladp0079s0134.2	754	kal-miR530a	0	0.12422	ATSRG1,SRG1
Kaladp0037s0196.1	545	kal-miR858	0	0.12497	TFIIS
Kaladp0016s0093.1	2414	kal-miR_C625_star	0	0.12723	
Kaladp0102s0125.1	463	kal-miR858	0	0.12873	AtMYB36,MYB36
Kaladp0042s0136.2	1097	kal-miR_C207_star	2	0.12954	ATMYB65,MYB65
Kaladp0036s0247.1	951	kal-miR_C5376	2	0.12954	
Kaladp0550s0075.1	986	kal-miR156a_star	2	0.12954	
Kaladp0043s0276.1	1293	kal-miR166a	2	0.12954	ATHB-15,ATHB15,CNA,ICU4
Kaladp0024s0202.1	243	kal-miR166a_star	2	0.12954	LTA2,PLE2
Kaladp0779s0018.5	217	kal-miR169c_star	2	0.12954	APG2,PGA2,TATC,UNE3
Kaladp0088s0122.1	366	kal-miR319	2	0.12954	
Kaladp0062s0033.1	1177	kal-miR530a_star	2	0.12954	CER6,CUT1,G2,KCS6,POP1
Kaladp0015s0067.1	1441	mdm-miR171c	2	0.12954	ATHAM3,HAM3
Kaladp0007s0058.1	181	vvi-MIR3627-p5_2ss5CG18CA	2	0.12954	ELF9
Kaladp0048s0183.2	568	kal-miR_C625_star	0	0.1336	SKIP6
Kaladp0048s0183.3	568	kal-miR_C625_star	0	0.13397	SKIP6
Kaladp0048s0183.1	568	kal-miR_C625_star	0	0.13434	SKIP6
Kaladp0808s0041.2	572	mdm-miR160a	0	0.13695	ATERF-5,ATERF5,ERF5
Kaladp0059s0142.2	969	ath-MIR5655-p5_2ss15CT18TA	3	0.13847	

Kaladp0048s0753.1	252	cca-miR396a-3p_L+2R-2	3	0.13847	
Kaladp0024s0688.1	687	kal-miR164d_star	3	0.13847	
Kaladp0048s0753.1	250	kal-miR396b_star	3	0.13847	
Kaladp0003s0155.1	888	mdm-miR162a	3	0.13847	
Kaladp0891s0016.1	1751	mdm-miR172i_L+1R-1	3	0.13847	AP2,FL1,FLO2
Kaladp0048s0753.1	249	stu-miR396-3p_L-1_1ss3CT	3	0.13847	
Kaladp0040s0498.1	399	mdm-miR159d	1	0.1428	
Kaladp0049s0025.4	424	mdm-miR393g_2ss12CT16TC	0	0.14766	TAPX
Kaladp0083s0083.2	77	kal-miR166a_star	0	0.15461	ATMAP2K_ALPHA,ATMEK5,ATMKK5,MAP2K_A,MEK5,MKK5
Kaladp0011s0995.1	1125	bol-MIR9410-p3_2ss5TG18TA	4	0.15574	
Kaladp0088s0122.3	366	kal-miR319	4	0.15574	
Kaladp0433s0005.1	1687	kal-miR_C625_star	0	0.15606	VHA-A
Kaladp0048s0113.1	846	mdm-MIR397a-p3_2ss7TG21AG	0	0.17475	AtVEX1
Kaladp0092s0167.1	338	mtr-MIR2673a-p3_2ss3TA20TG	0	0.18324	
Kaladp0067s0129.1	523	mdm-miR159d	0	0.18359	ATBPC6,BBR/BPC6,BPC6
Kaladp1222s0012.1	1379	fve-MIR845-p5_2ss6GC19AT	2	0.18788	
Kaladp0030s0092.1	199	ghr-MIR827a-p3_1ss6CG	2	0.18788	
Kaladp0024s0285.1	2321	kal-miR_C207_star	2	0.18788	ATMYB65,MYB65
Kaladp0095s0509.1	1302	kal-miR166a	2	0.18788	ATHB-15,ATHB15,CNA,ICU4
Kaladp0779s0018.3	215	kal-miR169c_star	2	0.18788	APG2,PGA2,TATC,UNE3
Kaladp0048s0043.1	125	kal-miR319_star	2	0.18788	ATL31,CNI1

Kaladp0072s0073.1	526	lus-MIR399b-p5_2ss6AG17AT	2	0.18788	
Kaladp0099s0085.4	1675	mdm-miR172i_L+1R-1	2	0.18788	RAP2.7,TOE1
Kaladp0003s0028.1	804	mdm-MIR397a-p3_2ss7TG21AG	2	0.18788	LAC12
Kaladp0048s0753.2	249	stu-miR396-3p_L-1_1ss3CT	2	0.18788	
Kaladp0076s0185.1	442	vvi-miR3630-3p_L-1_1ss2TA	2	0.18788	
Kaladp0059s0142.1	953	ath-MIR5655-p5_2ss15CT18TA	3	0.20034	
Kaladp0036s0141.1	938	cca-miR396a-3p_L+2R-2	3	0.20034	
Kaladp0102s0029.1	79	kal-miR_C400_star	3	0.20034	
Kaladp0060s0028.1	651	kal-miR_C625_star	3	0.20034	
Kaladp0068s0375.1	357	kal-miR_C689_star	3	0.20034	HXK3
Kaladp0008s0921.2	392	kal-miR172c_star	3	0.20034	
Kaladp0042s0225.1	456	kal-miR858	3	0.20034	
Kaladp0002s0088.1	1900	mdm-miR160a_L+1R-1	3	0.20034	ARF16
Kaladp0047s0031.1	568	mdm-miR168a_1ss2CT	3	0.20034	
Kaladp0110s0011.1	233	mdm-miR403a	3	0.20034	
Kaladp0048s0788.1	2721	kal-miR_C5376	4	0.20207	SEU
Kaladp0103s0028.1	853	kal-miR_C625_star	4	0.20207	EMB161,EMB251,EMB2775,RFC3
Kaladp0779s0018.1	217	kal-miR169c_star	4	0.20207	APG2,PGA2,TATC,UNE3
Kaladp0008s0921.1	392	kal-miR172c_star	4	0.20207	
Kaladp0058s0478.2	181	mtr-miR396b-3p	4	0.20207	GUX1,PGSIP1

Kaladp0152s0023.1	437	vvi-MIR3627-p5_2ss5CG18CA	1	0.23106	AtTLP2,TLP2
Kaladp0093s0071.1	388	nta-MIR171a-p5_1ss11GA	0	0.23334	
Kaladp0023s0071.2	530	fve-miR396e_R+1_1ss21TC	2	0.2423	AtGRF1,GRF1
Kaladp0103s0004.2	1556	gma-MIR5775-p3_2ss9AC18GT	2	0.2423	VAD1
Kaladp0042s0136.1	2092	kal-miR_C207_star	2	0.2423	ATMYB65,MYB65
Kaladp0053s0163.1	808	kal-miR164d_star	2	0.2423	AtXRN3,XRN3
Kaladp0092s0159.1	1343	kal-miR166a	2	0.2423	ATHB-15,ATHB15,CNA,ICU4
Kaladp0011s1053.1	844	mdm-miR156t	2	0.2423	
Kaladp0008s0410.1	1783	mdm-miR160a_L+1R-1	2	0.2423	ARF16
Kaladp0099s0085.2	1729	mdm-miR172i_L+1R-1	2	0.2423	RAP2.7,TOE1
Kaladp0082s0121.1	431	mdm-MIR397a-p3_2ss7TG21AG	2	0.2423	
Kaladp0084s0021.3	407	kal-miR_C689_star	4	0.24585	
Kaladp0043s0306.1	1131	kal-miR858_star	4	0.24585	AAA1,ATKTN1,BOT1,ERH3,FRA2,FRC2,FTR,KTN1,LUE1
Kaladp0072s0073.4	632	lus-MIR399b-p5_2ss6AG17AT	4	0.24585	
Kaladp0017s0020.2	1173	mdm-miR156t_R-1_1ss15AT	4	0.24585	
Kaladp0039s0588.1	586	bol-MIR9410-p3_2ss5TG18TA	3	0.25776	
Kaladp0095s0382.1	395	kal-miR_C400_star	3	0.25776	ATMAPK3,ATMPK3,MPK3
Kaladp0475s0002.1	398	kal-miR156a_star	3	0.25776	
Kaladp0088s0122.2	365	kal-miR319	3	0.25776	
Kaladp0072s	292	lus-MIR399b-	3	0.25776	

0073.2		p5_2ss6AG17AT			
Kaladp0085s 0097.1	335	mdm-miR403a	3	0.25776	
Kaladp0055s 0162.1	620	mdm-MIR397a-p3_2ss7TG21AG	0	0.2593	
Kaladp0010s 0061.1	639	ath-MIR858a-p5_1ss17AG	1	0.26609	
Kaladp0043s 0292.1	2260	kal-miR169c_star	1	0.26638	
Kaladp1221s 0044.1	846	kal-miR396b	0	0.28439	
Kaladp0006s 0047.1	85	kal-miR156a_star	4	0.28723	KING1
Kaladp0038s 0106.1	1550	kal-miR166a_star	4	0.28723	
Kaladp0062s 0137.1	1000	kal-miR167a	4	0.28723	ATLIP1,LIP1
Kaladp0067s 0257.3	305	kal-miR172c_star	4	0.28723	ARFA1F,ATARFA1F
Kaladp0011s 0278.2	1271	fve-miR396e_R+1_1ss21TC	2	0.29308	AtGRF3,GRF3
Kaladp0103s 0004.1	1563	gma-MIR5775-p3_2ss9AC18GT	2	0.29308	VAD1
Kaladp0095s 0382.2	395	kal-miR_C400_star	2	0.29308	ATMAPK3,ATMPK3,MPK3
Kaladp0008s 0827.3	1030	kal-miR166a	2	0.29308	ATHB-14,ATHB14,PHB,PHB-1D
Kaladp0779s 0018.4	217	kal-miR169c_star	2	0.29308	APG2,PGA2,TATC,UNE3
Kaladp0028s 0100.1	818	kal-miR319	2	0.29308	AtHsp90.2,ERD8,HSP81-2,HSP90.2
Kaladp0094s 0012.2	1561	mdm-miR156t	2	0.29308	
Kaladp0059s 0103.1	440	mdm-miR162a	2	0.29308	AtCTR1,CTR1,SIS1
Kaladp0099s 0085.1	1551	mdm-miR172i_L+1R-1	2	0.29308	RAP2.7,TOE1
Kaladp0048s 0753.2	335	mdm-miR396b_1ss2TC	2	0.29308	
Kaladp0048s 0652.1	2725	vvi-miR3630-3p_L-1_1ss2TA	2	0.29308	

Kaladp0011s0186.1	870	kal-miR_C625_star	3	0.31107	
Kaladp0016s0260.1	482	kal-miR167a	3	0.31107	
Kaladp0068s0032.1	707	kal-miR397d	3	0.31107	ATLAC1,LAC1
Kaladp0674s0024.1	733	kal-miR858	3	0.31107	
Kaladp0039s0575.1	1602	mdm-miR160a_L+1R-1	3	0.31107	ARF16
Kaladp0051s0089.1	229	mdm-miR403a	3	0.31107	AtHsp90.4,Hsp81.4
Kaladp0048s0102.5	211	csi-MIR167b-p3_1ss19CT	4	0.32634	
Kaladp0090s0069.2	1121	kal-miR397d_star	4	0.32634	FAB1B
Kaladp0101s0088.2	698	kal-miR530a_star	4	0.32634	AT-HSFB2A,HSFB2A
Kaladp0008s0029.2	284	mdm-MIR10981b-p5_2ss15TG18AT	4	0.32634	
Kaladp0808s0002.1	596	ath-MIR5655-p5_2ss15CT18TA	2	0.34045	
Kaladp0048s0102.6	211	csi-MIR167b-p3_1ss19CT	2	0.34045	
Kaladp0023s0071.1	1072	fve-miR396e_R+1_1ss21TC	2	0.34045	AtGRF1,GRF1
Kaladp0041s0017.1	244	fve-MIR845-p5_2ss6GC19AT	2	0.34045	
Kaladp0021s0092.1	1440	kal-miR_C5376	2	0.34045	
Kaladp0039s0699.2	814	kal-miR166a	2	0.34045	IFL,IFL1,REV
Kaladp0032s0009.1	787	kal-miR166a_star	2	0.34045	ATSCO1,ATSCO1/CPEF-G,SCO1
Kaladp0096s0089.1	756	kal-miR169c	2	0.34045	
Kaladp0779s0018.2	215	kal-miR169c_star	2	0.34045	APG2,PGA2,TATC,UNE3
Kaladp0094s	1364	mdm-	2	0.34045	

0012.3		miR156t_L-1_1ss15AT			
Kaladp0032s0311.1	1526	mdm-miR172i_L+1R-1	2	0.34045	RAP2.7,TOE1
Kaladp0056s0149.1	1451	kal-miR164d_star	3	0.36054	TSL
Kaladp0048s0789.1	565	kal-miR167a_star	3	0.36054	
Kaladp0606s0013.1	1074	kal-miR319	3	0.36054	CLT3
Kaladp0043s0253.1	328	kal-miR319_star	3	0.36054	CARA
Kaladp0043s0306.2	998	kal-miR858_star	3	0.36054	AAA1,ATKTN1,BOT1,ERH3,FRA2,FRC2,FTR,KTN1,LUE1
Kaladp0059s0103.3	421	mdm-miR162a	3	0.36054	AtCTR1,CTR1,SIS1
Kaladp0048s0753.1	335	mdm-miR396b_1ss2TC	3	0.36054	
Kaladp0001s0295.1	1302	hbr-MIR6173-p5_2ss21AG22GC	4	0.3633	
Kaladp0090s0069.1	1255	kal-miR397d_star	4	0.3633	FAB1B
Kaladp0008s0029.3	284	mdm-MIR10981b-p5_2ss15TG18AT	4	0.3633	
Kaladp0051s0086.2	325	mdm-miR168a_1ss2CT	4	0.3633	
Kaladp0091s0175.2	127	mdm-miR2111a	4	0.3633	
Kaladp1222s0058.1	666	aly-MIR408-p5_2ss4CT20TC	1	0.37077	
Kaladp0011s0278.1	1271	fve-miR396e_R+1_1ss21TC	2	0.38465	AtGRF4,GRF4
Kaladp0047s0013.1	2255	fve-MIR845-p5_2ss6GC19AT	2	0.38465	BGAL3
Kaladp0011s0987.1	1602	kal-miR_C5376	2	0.38465	ATAZG1,AZG1
Kaladp0032s0009.2	787	kal-miR166a_star	2	0.38465	ATSCO1,ATSCO1/CPEF-G,SCO1

Kaladp0003s0134.2	1083	kal-miR858	2	0.38465	
Kaladp0011s0688.1	101	kal-miR858_star	2	0.38465	PRA1.A2
Kaladp0059s0103.2	440	mdm-miR162a	2	0.38465	AtCTR1,CTR1,SIS1
Kaladp0037s0298.1	637	mdm-miR168a_1ss2CT	2	0.38465	
Kaladp0032s0311.3	1496	mdm-miR172i_L+1R-1	2	0.38465	RAP2.7,TOE1
Kaladp0091s0175.1	127	mdm-miR2111a	2	0.38465	
Kaladp0042s0242.1	989	mdm-miR396b_1ss2TC	2	0.38465	
Kaladp0055s0456.2	776	kal-miR_C1878_star	4	0.39824	
Kaladp0067s0271.1	599	kal-miR164d	4	0.39824	ATUXS2,AUD1,UXS2
Kaladp0053s0164.1	694	kal-miR172c_star	4	0.39824	APS2,ASA1
Kaladp0091s0175.4	127	mdm-miR2111a	4	0.39824	
Kaladp0011s0106.1	984	hbr-MIR6173-p5_2ss21AG22GC	3	0.40646	
Kaladp0023s0025.2	877	kal-miR169c	3	0.40646	ATHMA1,HMA1
Kaladp0091s0111.1	385	kal-miR319_star	3	0.40646	
Kaladp0085s0126.1	168	nta-MIR168a-p3_2ss1CA19GC	3	0.40646	
Kaladp0031s0011.1	596	ath-MIR5655-p5_2ss15CT18TA	2	0.42589	
Kaladp0022s0101.2	761	kal-miR_C450	2	0.42589	ATLCB1,EMB2779,FBR11,LCB1
Kaladp0040s0482.1	919	kal-miR_C450_star	2	0.42589	
Kaladp0008s0827.2	1059	kal-miR166a	2	0.42589	ATHB-14,ATHB14,PHB,PHB-1D
Kaladp0101s	815	kal-	2	0.42589	AT-HSFB2A,HSFB2A

0088.1		miR530a_star			
Kaladp0003s 0134.3	799	kal-miR858	2	0.42589	ATMYB12,MYB12,PFG1
Kaladp0094s 0012.2	1560	mdm-miR156t_L-1_1ss15AT	2	0.42589	
Kaladp0048s 0026.1	1911	mdm-miR160a_L+1R-1	2	0.42589	ARF16
Kaladp0042s 0133.2	2035	vvi-MIR3627-p5_2ss5CG18CA	1	0.42713	
Kaladp0550s 0061.2	1741	gma-MIR5368-p3_2ss1AT18CA	4	0.43126	
Kaladp0055s 0456.1	990	kal-miR_C1878_star	4	0.43126	
Kaladp0068s 0181.1	190	kal-miR_C689_star	4	0.43126	
Kaladp0095s 0221.1	2184	kal-miR172c_star	4	0.43126	ATCAP-C,ATSMC3,ATSMC4,SMC3
Kaladp0012s 0053.1	1257	gma-MIR5775-p3_2ss9AC18GT	3	0.44909	
Kaladp0021s 0108.1	1121	kal-miR_C207	3	0.44909	ATWL4,CIPK12,SnRK3.9,WL4
Kaladp0067s 0271.2	599	kal-miR164d	3	0.44909	ATUXS2,AUD1,UXS2
Kaladp0011s 0056.1	459	kal-miR858_star	3	0.44909	ATMEPCT,ISPD,MCT
Kaladp0740s 0004.1	1965	lja-miR397_L-2R+2	3	0.44909	
Kaladp0032s 0311.2	1451	mdm-miR172i_L+1R-1	3	0.44909	RAP2.7,TOE1
Kaladp0085s 0126.2	100	nta-MIR168a-p3_2ss1CA19GC	3	0.44909	
Kaladp0020s 0210.2	1316	gma-MIR5775-p3_2ss9AC18GT	4	0.46247	
Kaladp0048s 0514.7	376	mdm-miR403a	4	0.46247	PSD
Kaladp0048s 0892.1	2564	ath-MIR858a-p5_1ss17AG	0	0.46398	
Kaladp0023s 0084.1	297	bol-MIR9410-p3_2ss5TG18TA	2	0.46437	HSL2
Kaladp0032s	977	fve-	2	0.46437	AtGRF5,GRF5

0085.1		miR396e_R+1_1ss21TC			
Kaladp0053s0155.1	194	gma-MIR5775-p3_2ss9AC18GT	2	0.46437	
Kaladp0021s0108.2	1125	kal-miR_C207	2	0.46437	ATWL4,CIPK12,SnRK3.9,WL4
Kaladp0095s0791.1	289	kal-miR_C5376	2	0.46437	TBL34
Kaladp0081s0257.1	1751	kal-miR166a	2	0.46437	IFL,IFL1,REV
Kaladp0093s0040.2	2113	kal-miR397d	2	0.46437	
Kaladp0003s0134.4	992	kal-miR858	2	0.46437	
Kaladp0746s0002.1	865	mdm-miR156t_R-1_1ss15AT	2	0.46437	SPL3
Kaladp0095s0416.1	273	mdm-miR162a	2	0.46437	
Kaladp0071s0243.2	1752	mdm-miR172i_L+1R-1	2	0.46437	RAP2.7,TOE1
Kaladp0022s0101.1	1025	kal-miR_C450	3	0.48865	ATLCB1,EMB2779,FBR11,LCB1
Kaladp0048s0887.1	498	kal-miR167a	3	0.48865	
Kaladp0023s0025.1	877	kal-miR169c	3	0.48865	ATHMA1,HMA1
Kaladp0093s0040.3	2279	lja-miR397_L-2R+2	3	0.48865	
Kaladp0007s0071.2	881	lus-MIR399b-p5_2ss6AG17AT	3	0.48865	
Kaladp0051s0086.1	404	mdm-miR168a_1ss2CT	3	0.48865	
Kaladp0042s0159.1	334	ppe-MIR171e-p5	3	0.48865	NQR
Kaladp0039s0644.1	776	kal-miR_C516_star	4	0.49196	
Kaladp0008s0481.1	571	kal-miR397d_star	4	0.49196	
Kaladp0024s0978.1	202	kal-miR530a_star	4	0.49196	FBA2

[1] Gehrig H, GauβMann O, Marx H, Schwarzottb D, Klugeb M. **Molecular phylogeny of the genus Kalanchoe (Crassulaceae) inferred from nucleotide sequences of the ITS-1 and ITS-2 regions**. *Plant Science*, 2001, **160**(5):827-835.
